# Epigenetic perturbations in aging stem cells

**DOI:** 10.1007/s00335-016-9645-8

**Published:** 2016-05-26

**Authors:** Sara Russo Krauss, Gerald de Haan

**Affiliations:** Department of Ageing Biology and Stem Cells, European Research Institute for the Biology of Ageing, University Medical Centre Groningen, University of Groningen, Antonius Deusinglaan 1, 9713 AV Groningen, The Netherlands

## Abstract

Stem cells maintain homeostasis in all regenerating tissues during the lifespan of an organism. Thus, age-related functional decline of such tissues is likely to be at least partially explained by molecular events occurring in the stem cell compartment. Some of these events involve epigenetic changes, which may dictate how an aging genome can lead to differential gene expression programs. Recent technological advances have made it now possible to assess the genome-wide distribution of an ever-increasing number of epigenetic marks. As a result, the hypothesis that there may be a causal role for an altered epigenome contributing to the functional decline of cells, tissues, and organs in aging organisms can now be explored. In this paper, we review recent developments in the field of epigenetic regulation of stem cells, and how this may contribute to aging.

## Introduction

Aging is associated with a progressive decline in function of adult tissues and organs observed in all mammals. Adult stem cells have now been characterized in almost all mammalian tissues, including blood, skeletal muscle, intestine, skin, and brain. These tissue-specific stem cells possess self-renewal potential and the ability to generate mature cells: characteristics they need in order to maintain tissue homeostasis and regeneration of the tissue after stress or cell loss. Within many aged tissues, a loss of the regenerative capacity of adult stem cells has been documented. Therefore, impaired stem cell function, more than intrinsic changes in differentiated cells, has been considered as a driver of the aging process of multiple regenerating tissues, and as such may contribute to organismal aging. Such stem cell-intrinsic events could theoretically involve either genetic or epigenetic changes. Whereas the role of an accumulation of genetic lesions in stem cell functioning during aging has been recently reviewed elsewhere (Behrens et al. 2014), in the current manuscript we focus on the role of age-associated epigenetic changes. “Epigenetics” is a term used to classify heritable changes of gene expression that are not attributed to changes in the DNA sequence (Goldberg et al. 2007). Due to the fundamental role of epigenetics in the regulation of gene expression and the putative reversibility of such epigenetic marks, there is an increasing interest in the role of epigenetic processes as mediators of the aging process of stem cells. In this review, we discuss the biology of stem cell aging with a particular focus on the epigenetic contribution to the aging process. We briefly explain current methods to evaluate epigenetic marks in the context of biological aging and discuss to what extent these have revealed a common epigenetic pattern in stem cell aging.

## Do aging stem cells contribute to the functional decline of organs?

As individuals age, there is a gradual loss of homeostasis of most tissues and, as a consequence, a decline in organ function. A large body of data suggests that in many tissues age-associated loss of homeostasis is caused by an age-related decline in the ability of stem cells to replace damaged cells, (reviewed in Rando 2006; Drummond-Barbosa 2008; Liu and Rando 2011). For example, skeletal muscle possesses remarkable regenerative ability upon injury, a process that is mediated by the resident muscle stem cells. However, muscle stem cells isolated from aged animals have a higher propensity to undergo fibrogenic differentiation (Brack et al. 2007). As a result, upon aging there is an increase in tissue fibrosis and the subsequent aged-related reduction in the mass of muscle tissue contributes to an impaired motor activity in the elderly. Similarly, aging in the nervous system leads to the loss of neuronal stem cells (NSCs) (Molofsky et al. 2006). NSCs in the adult brain give rise to new granule layer neurons that integrate into functional neuronal circuits (Song et al. 2002), supporting processes such as learning and memory formation (Clelland et al. 2009), which are often impaired as individuals age. Also in the skin, melanocyte stem cells that pigment new hair drop in number upon aging (Maslov et al. 2004), leading to the very common phenotype observed in the elderly, hair loss and graying (Nishimura et al. 2005). However, in mammals, not every organ is directly dependent on stem cell activity. Aging-related alterations in organs like eyes, inner ears, or bones are more difficult to attribute to impaired stem cell activity. Retinal stem cells can potentially account for age-related diseases like macular degeneration, but not for the changes in corneal curvature or in the condensation of the vitreous gel that cause alteration in refraction and decreased sight capacity in elderly. Similarly, ear sensory cells do not regenerate if lost (Groves 2010); therefore, aged-associated loss of hearing has so far not been associated to stem cell exhaustion.

Understanding the basic properties of the various types of tissue-specific stem cells and cataloguing the molecular changes that accumulate in these cells as they age is of great interest. In particular, insight into molecular changes that could potentially be reversible, such as epigenetic alterations, may open options to develop therapeutic approaches for age-related diseases based on interventions to delay or prevent stem cell aging.

## Functional and molecular manifestations of stem cell aging

In the section above, we introduced the aged-associated decline of function at the level of tissues and organs. In the following sections, we discuss the main functional manifestation and molecular changes that occur in several regenerating tissues as stem cells age. In particular, we will focus on age-related changes that appear to occur commonly versus changes that are tissue specific.

### Stem cell pool size

In almost all mammalian tissues that are capable to regenerate, the number of adult stem cells is affected by aging. However, the directionality of this change is variable. Stem cells in the hematopoietic tissue have been reported to increase in number (de Haan and Van Zant 1999), and this age-dependent expansion of HSCs is a transplantable, cell-intrinsic aspect of hematopoietic stem cells. On the contrary, skin and muscle stem cells display an age-dependent decrease in number (Nishimura et al. 2005; Renault et al. 2002). In brain, changes in the neuronal stem cell pool appear to be region-specific (Kuhn et al. 1996; Maslov et al. 2004; Hattiangady and Shetty 2008). Currently, we know very little about how stem cell pool size in these various tissues is actually regulated. However, despite the diverse directionality of the change in stem cell pool size, alterations in the numbers of stem cells during aging suggest that deregulation of self-renewal and cell fate programs, i.e., a loss of control of stem cell pool size, might be a common molecular event occurring during aging. It is worth considering, however, that measurements of stem cell compartment size is confounded by the current inability to purify stem cells to homogeneity, as well as the lack of adequate models to test the function of many stem cell types.

### Impaired functionality and altered lineage commitment

The decline in stem cell functionality is a shared feature among the majority of adult stem cell compartments. Defects of aged HSCs in long-term reconstitution of the immune system have been demonstrated in competitive transplantation assays (Kamminga et al. 2005; Rossi et al. 2005). Neurogenesis potential of neuronal stem cells in the nervous system declines with age (Bondolfi et al. 2004; Enwere et al. 2004). Similarly, aged muscle satellite cells display impaired muscle regeneration after injury (Conboy et al. 2003; Carlson and Conboy 2007). The loss of functionality in a stem cell compartment directly translates into a decline in the function of progenitors cells and ultimately into an altered differentiation program. Thus, an aging-associated feature shared among many adult stem cell compartments is the aberrant lineage specification of stem cell progeny. Within the hematopoietic system, stem cells from both old humans and old mice show an increased propensity to differentiate along the myeloid, rather than the lymphoid lineage (Sudo et al. 2000; Rossi et al. 2005; Dykstra et al. 2007; Cho et al. 2008). In the brain, there is an increased production of astrocytes (astroglial lineage skewing) (Peinado et al. 1998). Aged muscles are characterized by the increased tendency of satellite cells to convert from myogenic to fibroblastic and adipogenic lineages (Taylor-Jones et al. 2002; Brack et al. 2007). The accumulation of abnormal progenies in all the tissues reported above critically contributes to the gradual deterioration of tissue structure and function associated with aging.

### Molecular alterations underling stem cell aging

Several molecular changes, including perturbation of signaling pathways, mitochondrial dysfunction, metabolic and cell energetic deregulation, have been implicated in tissue-specific aging patterns and have been extensively reviewed elsewhere (Lopez-Otin et al. 2013). In general, cell-intrinsic changes can be broadly grouped into two classes: those that are genetic, and therefore typically irreversible, versus those that are epigenetic. The extent to which mostly irreversible genomic changes (including nuclear and mitochondrial DNA damage and telomere shortening) contribute to stem cell aging is an active area of investigation and has been extensively reviewed elsewhere (Adams et al. 2015). Mouse models involving DNA repair proteins have shown that an aging-like phenotype is accelerated in the presence of increased DNA damage (reviewed in Garinis et al. 2008). However, so far no experimental evidence has been provided which demonstrates that repressing the occurrence of DNA mutations leads to an extension of life span.

DNA mutations can also occur in genes coding for epigenetic modifiers, leading to an altered epigenetic regulation and possibly altered fate programs. Interestingly, common DNMT3A mutations have been recently described to arise early in the development of acute myeloid leukemia, a type of leukemia that preferentially occurs in the elderly. DNMT3a mutations appear to lead to a clonally expanded pool of pre-leukemic stem cells from which AML evolves (Shlush et al. 2014).

Whereas most genetic changes that occur in somatic stem cells are believed to be irreversible, molecular changes that affect epigenetic moieties are potentially reversible. What are the indications that such epigenetic changes are associated with stem cell aging?

## Epigenetic control of stem cell regulation

**Fig. 1:**
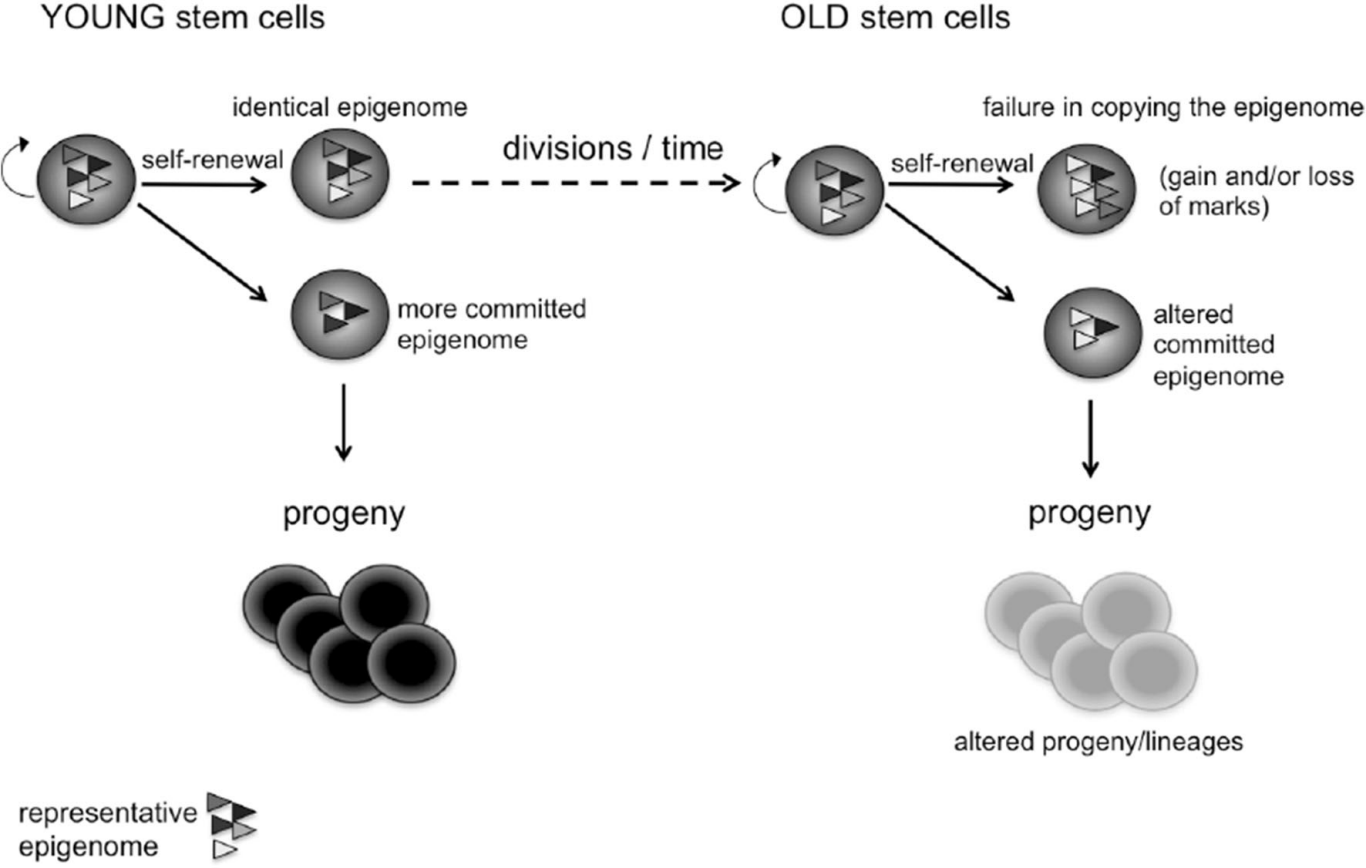
Epigenetic alterations in aging of stem cells. Upon multiple cell divisions that occur as a function of time, infidelity in exact replication of the epigenome to daughter cells might occur. Both detrimental losses and gains of epigenetic modifications can thus be amplified within the stem cell pool and propagated to the progeny upon differentiation

Epigenetics is the study of phenotypes or gene expression patterns that are heritable through cell divisions, but remain independent of DNA sequence (Berger et al. 2009; Goldberg et al. 2007; reviewed in Bonasio et al. 2010). Epigenetic regulation is required for the establishment and maintenance of biological states (Grewal and Klar 1996; Cavalli and Paro 1998; Martin and Zhang 2007). Thus, while in differentiated-end stage-cells epigenetic regulation is mainly used to regulate ongoing cellular processes, in stem cells the implications of the epigenome are wider. During a self-renewing division, a stem cell must not only replicate its genome flawlessly but also must copy all the relevant epigenetic markers, deposited by a large number of writers and erasers, to at least one of the two daughter cells, in the timeframe of a single cell division. Epigenetic regulation in adult stem cells must ensure the co-occurrence of self-renewal programs together with instructions for differentiation into cells with distinct potentials and, in some instances, into a large set of mature cells of different lineages with vast functional heterogeneity. Thus, epigenetic alterations that arise in stem cells can be amplified through self-renewal programs and propagated to the progeny upon differentiation. Infidelity in the epigenetic stem cell machinery in stem cells could result in detrimental losses or gains of a plethora of marks during aging (Fig. 1).

### DNA methylation

Epigenetic regulation can occur at different levels. At the DNA level, epigenetic modification is shaped by the addition or removal of a methyl group or hydroxymethyl group at the 5th carbon atom of cytosine. DNA methyltransferase enzymes are responsible for both the establishment (DNMT3A and DNMT3B) and maintenance (DNMT1) of methylated nucleotides. Many studies have documented a role for these enzymes in balancing self-renewal and differentiation in multiple adult stem cell compartments (reviewed in Beerman and Rossi 2015). The aberrant stem cell functioning caused by dysregulation of DNA methylation leads to phenotypes frequently mirroring those observed with aging. Loss of Dnmt1 in HSCs leads to dysregulation of lineage output, with a skewing towards myelopoiesis (Broske et al. 2009), a phenotype largely associated with HSCs aging. Similarly, Dnmt3a ablation in HSCs predisposes mice to develop a spectrum of myeloid and lymphoid malignancies, and Dnmt3a-KO-derived myeloid malignancies and T cell acute lymphocytic leukemia/lymphoma show distinct methylation aberrations (Mayle et al. 2015). Interestingly, Dnmt3a has been found to be slightly but significantly downregulated with age (Sun et al. 2014).

The gold standard for direct analysis of DNA methylation is the sequencing-based quantification of cytosine methylation following bisulfite conversion, which allows identification and quantification of DNA methylation at single nucleotide resolution. A description of current techniques and methods used for analysis of DNA methylation has been recently reported (Plongthongkum et al. 2014). An ever-increasing effort has been made towards the application of assays aimed to quantify DNA methylation in a limited amount of input material (Table 1), such as highly purified populations of stem cells. Experiments that use low numbers of cells are predicted to reduce bias originating from cell-to-cell variability, even within a relatively homogeneous cell population. Early studies examined global DNA methylation patterns in aged tissues, and largely demonstrate age-associated hypo-methylation (Wilson and Jones 1983; Wilson et al. 1987; Heyn et al. 2012; Florath et al. 2014; Jung and Pfeifer 2015). Nonetheless, global DNA hypo-methylation does not exclude the possibility that individual loci become hypermethylated during aging. Indeed, along with a genome-wide analysis of the global DNA methylation landscape, several studies report have uncovered locus-specific DNA hyper-methylation associated with aging tissues (Maegawa et al. 2010; Rakyan et al. 2010; Zykovich et al. 2014). Interestingly, DNA methylation of a limited set of specific loci has been hypothesized to provide biomarkers for the aging process (Horvath 2013; Weidner et al. 2014). It is worth noticing that studies in neuronal, hematopoietic, and skin stem cells in which the DNA methylome was correlated with the transcriptome revealed that altered DNA methylation profiles not always directly correlated with altered transcription in stem cells (Guo et al. 2011; Bock et al. 2012; Beerman et al. 2013; Sun et al. 2014).

**Table 1:** Analysis of the epigenome: recent methods for single cell or ultra-low-input assays

Assay	Developer	Input material
Nano-ChIP-seq	Adli and Bernstein 2011	10,000 cells for H3k4me3 histone mark
Linear DNA amplification (LinDA)	Shankaranarayanan et al. 2011	For transcription factors using 5000 cells and for the H3K4me3 histone modification using 10,000 cells
Ultra-low-input micrococcal nuclease-based native ChIP (ULI-NChIP)	Brind’Amour et al. 2015	10,000 for histone marks
ChIPmentation	Schmidl et al. 2015	For several histone marks 10,000 cells per IP, and 100,000 cells for transcription factors
Single-cell ChIP-seq	Rotem et al. 2015	One cell. ChIP-seq for H3k4me3 and H3k4me2
Single-cell reduced-representation bisulfite sequencing scRRBS	Guo et al. 2015	One cell. Non-targeted enrichment DNA methylation analysis
Low-input and single-cell whole-genome bisulfite sequencing (μWGBS, scWGBS)	Farlik et al. 2015	High-throughput bisulfite sequencing assay for low-input and single-cell samples
Single-cell bisulfite sequencing scBS-seq	Smallwood et al. 2014	One cell. Targeted enrichment DNA methylation analysis

### Histone variants, histone modifications, and higher order chromatin structure

Core histones proteins within a nucleosome can be substituted by several variants. Each variant histone contains specific physical properties, which differentially regulate expression of nearby DNA sequences. Very little is known about the contribution of different histone variants to the aging process. Beyond different histone variants, a next layer of epigenetic regulation occurs at histone tails that can be modified by an array of post-translational modifications, including acetylation, methylation, ubiquitylation, phosphorylation, sumoylation, ribosylation, and citrullination. These post-translational histone modifications affect transcription both directly, through changes in higher order chromatin structures, and indirectly, by recruitment of downstream effectors. Current techniques to analyze the modification of the histone tails and their implication in the aging process will be discussed into more detail in the next section. The combined effect of all these epigenetic mechanisms ultimately alters the higher order structure of the chromatin itself and the position of chromatin in the cell nucleus, allowing the recruitment or dismissal of transcription factors.

Substantial efforts have been made to understand the higher, three-dimensional genome organization within the nucleus. Chromosome conformation capture allows to analyze physical contacts between different regions of a chromosome and between the different chromosomes in cell populations (Dekker et al. 2002). In the past year, 4C, 5C, and Hi-C techniques have added new layers of information to the structure of chromosomes (reviewed in Cattoni et al. 2015). The development of single-cell approaches to reach single-cell, high-throughput, and high-resolution maps will be of great relevance as to what extent the 3-dimensional chromatin architecture in a nucleus of an aged (stem) cell is different from a young cell, and if so, whether or not this contributes to impaired stem cell functioning.

A third layer of epigenetic information is provided by nucleosome positioning and nucleosome occupancy. Recently, data have suggested that the variation in the total number of histone proteins may be altered during aging. The expression of core histones has been found to be reduced during replicative aging in yeast (Feser et al. 2010), as well as in aged muscle stem cells (Liu et al. 2013). Interestingly, increasing the cellular supply of histones increased the replicative lifespan of yeast by up to 50 % (Feser et al. 2010). The molecular mechanisms underlying this process, the genome-wide consequences of reduced nuclear histone content, and the mechanisms by which core histone expression levels are transferred to the daughter cells are still to be elucidated. Nonetheless, it is interesting to hypothesize that histone availability may have direct consequences on the epigenetic landscape of histone modifications and on transcription factor activity, as they compete with nucleosomes for binding to DNA.

### Epigenetic regulation by histone modifications

We will now focus in more detail on the epigenetic regulation by the spatial–temporal chemical modifications at the histone tails, and how these change upon aging.

Before the advent of genome-wide techniques to study the epigenome, research had been mainly focused on investigating whether specific histone modifications globally decreased or increased upon aging. These studies typically used Western blotting or immunostaining in order to directly quantify the amount of specific histone modifications in a given cell population. However, with this approach no information can be retrieved concerning differentially affected loci. Chromatin immunoprecipitation followed by high-throughput sequencing (ChIP-seq) is nowadays the standard technique for identifying genome-wide loci and biochemical modifications of chromatin-bound proteins and histone modifications. Promoters can feature multiple activating and repressive marks, although only few are typically assessed in a single experiment. Together with promoters, distal enhancers refine timing and extent of gene expression. Active or repressive enhancers are marked by specific chromatin moieties that can be identified in ChIP-seq analyses.

Currently, there is great interest in profiling enhancer-associated chromatin features in order to produce genome-wide maps of regulatory elements of clusters of genes. An outstanding contribution to this field has recently been provided by the Encyclopedia of DNA Elements (ENCODE), a public research project launched by the US National Human Genome Research Institute (NHGRI) in September 2003 (Feinglod Science 2004). As a follow-up of the Human Genome Project, ENCODE is ultimately dedicated to identify all functional elements in the human genome. In the Roadmap Epigenomics Project, epigenomic information has now been gathered for 127 cell types from most human tissues (Farh et al. 2015; Gjoneska et al. 2015; Leung et al. 2015; Polak et al. 2015; Roadmap Epigenomics et al. 2015; Ziller et al. 2015). This has resulted in the identification of hundreds of thousands of enhancer-like regions in the mammalian genome that regulate gene expression at long range. Although at present it is not yet feasible to determine how many features are required for a minimal representation of the epigenome, the consortium has focused so far on DNA methylation, six histone modifications (H3K4me1, H3K4me3, H3K9me3, H3K9ac, H3K27me3, and H3K36me3), and chromatin accessibility. The emerging and may be not too surprising picture is that combinations of multiple modifications predict gene activity in ways that a single type of modification does not.

Although these data provide a massive amount of epigenetic information in a large set of human cell types, it is important to notice that these experiments, and indeed most studies, are still based on ChIP-seq protocols that require a large numbers of cells (~10e6) as starting material. This limitation precludes the analysis of rare primary stem cell populations. Therefore, in recent past, attempts have been made to optimize new protocols suitable for low-input ChIP-seq. Some of the recent methods for single cell or ultra-low-input assay for the study of histone modifications and DNA methylation are summarized in Table 1. Challenges that need to be overcome relate to the fact that PCR amplification from limited ChIP input material more easily leads to amplification artifacts and duplicate reads, and to a reduced complexity of the immunoprecipitated material recovered. In addition, different bioinformatic approaches are used to analyze and present data, which ultimately results in substantial discordance of conclusions between seemingly similar studies generated from different laboratories.

## Do histone modifications alter during stem cell aging?

As mentioned in the previous sections, the direct analysis of histone modification in rare adult stem cell populations during aging has been challenging for a long time, as most of chromatin IP protocols require a high number of cells as a starting material. Consequently, as of to date only two recent studies have addressed whether an altered epigenetic regulation of histone modifications in mammalian stem cells is associated with aging. However, although this review focuses on epigenetic profiling in stem cell aging in mammals, it is important to consider that global loss or gain of histone modifications during aging have been reported in other model organisms (reviewed in Benayoun et al. 2015).

Recently, Sun et al. profiled two chromatin marks associated with active transcription, H3K4me3 and H3K36me3, and the repressive mark H3k27me3 in young and old HSCs using low-input ChIP-seq (Sun et al. 2014). Their analysis revealed that old HSCs exhibited a slight increase in the genome-wide number of H3K4me3 peaks, although no differences in expression levels of the various H3K4 methyltransferases were detected. Also, some H3K4me3 domains became broader upon aging. Interestingly, broad H3K4me3 domains have been reported to mark genes that are important for cell identity (Benayoun et al. 2014). Therefore, spreading of H3K4me3 during HSCs aging could account for the increase self-renewal and loss of lineage differentiation observed in aged HSCs. In contrast to what has been observed in HSCs, examination of the same H3K4me3 mark in muscle satellite cells showed few or no differences between cells isolated from young or aged mice (Liu et al. 2013).

The repressive Polycomb-mediated H3k27me3 mark, aged HSCs showed a similar number of peaks in aged compared to young HSCs, but these peaks appeared to become broader and more intense upon aging (Sun et al. 2014). This same effect was also observed in old muscle stem cells (Liu et al. 2013), both at the TSS and in intergenic regions. It is interesting to note that while the increase in H3k27me3 in activated versus quiescent muscle stem cells in young mice correlated with an increase in the expression of the H3K27 methyltransferase Ezh2, the accumulation of H3k27me3 in aging did not correlated with changes in the expression of neither Ezh2 nor with the H3K27 demethylase Jmjd3. Also, over 30 % of the genes that acquired H3K27me3 at their TSSs with age were not expressed in either young or old muscle stem cells, suggesting that the gain of the repressive H3K27me3 mark can underlie other mechanisms different from transcription suppression, and might be driven by processes that do not include canonical methyltransferases or demethylases (for example, decreased histone turnover and preferential accumulation of some histone marks).

As pointed out before, it is important to note that at present it is not trivial to cross-compare ChIP-Seq datasets generated from different groups, due to the huge variety of algorithms and custom-made pipelines. While commonly used peak calling algorithms identify whether a locus is being covered or not by a specific histone marks, it has become relevant also to use pipelines capable of identifying the extent to which individual loci are covered by different histone marks. Due to these issues, and due to the fact that at present only two studies have reported ChIP-seq profiles in aged stem cells (Sun et al. 2014; Liu et al. 2013), it is premature to conclude whether or not a common epigenetic stem cell signature exists in aged stem cells from different tissues.

## Mouse models to unravel the mechanism of epigenetic regulation

Next to biochemical assays to directly study chromatin modifications, epigenetic regulation in stem cells can be investigated using animal models in which enzymes responsible for the deposition or removal of histone marks (chromatin modifiers) are disrupted. This review will now focus on some of the most relevant findings generated from these studies.

The H3K27 methylation mark is added by the Polycomb repressive Complex 2 (PRC2), a complex that includes EED, SUZ12, and the catalytically active subunits EZH2/1. Alterations in expression enzymes involved in the transfer or removal of the methyl group on the lysine 27 of histone H3 have been shown to modulate organismal longevity in models other than mouse (reviewed in Benayoun et al. 2015; Jin et al. 2011; Maures et al. 2011; Ni et al. 2012; Siebold et al. 2010). In mammals, no studies have been reported that show that lifespan can be extended by perturbation of expression of epigenetic enzymes, but the effect of such proteins on (lifespan of) adult stem cells is well established. Most of our knowledge on the role of the PcG complexes in stem cells is based on studies in HSCs. For example, the PRC1 subunit Bmi-1 was shown to play an essential role in the generation of self-renewing adult HSCs (Park et al. 2003). Similarly, the Cbx7-PcG subunit was shown to regulate the balance between self-renewal and differentiation (Klauke et al. 2013). Overexpression of Ezh2 in HSCs prevents stem cell exhaustion during serial transplantation (Kamminga et al. 2006). A similar role of Ezh2 has been recently described in muscle satellite cell self-renewal (Woodhouse et al. 2013). Conversely, loss of Ezh1 induces significant depletion of adult HSCs (Hidalgo et al. 2012).

Whereas the Polycomb group proteins are predominantly involved in histone methylation and lead to gene repression, histone acetylation, which is associated with transcriptional activation, is controlled by the opposing activities of histone acetyltransferases (HATs) and histone deacetylases (HDACs). Although direct manipulation of HAT, and HDACs in stem cell with regard to age has not been reported yet, both HATs and HDACs have been implicated in modulating lifespan in mammals. Recent studies in transgenic mice overexpressing the histone deacetylase Sirtuin-1 in the brain showed significant life span extension, supporting a role for histone acetylation in health and lifespan (Satoh et al. 2013). Also, a role of the deacetylase Sirt6 in regulating mammalian longevity has been demonstrated. Mice carrying a constitutive deletion of Sirt6 show reduced lifespan and premature aging, while the overexpression of Sirt6 significantly increased lifespan in male mice (Mostoslavsky et al. 2006; Kanfi et al. 2012). The major mechanism of action of the HDACs is the deacetylation of the histone tails and the consequent transcriptional repression. However, it has also been shown that HDACs may exert their pro-longevity role by promoting increased genomic stability (Oberdoerffer et al. 2008; Wang et al. 2008; Toiber et al. 2013; Van Meter et al. 2014). A link between HATs and aging has also been provided in a progeroid mouse model (HGPS) where the decreased association of HATs with the nuclear periphery showed decreased H4K16ac and decreased lifespan (Krishnan et al. 2011). Interestingly, low H4K16ac levels have been correlated with aging in mouse hematopoietic stem cells (Florian et al. 2012). To what extent these lifespan changes result from stem cell-specific effects remains to be determined. It would therefore be of great interest to further investigate the role of HATs and HDACs during the aging of the multiple types of adult stem cells, especially as a potential therapeutic intervention aimed to selectively target these enzymes could contribute to prevention of aging-related stem cell impairment, and possibly organismal aging.

## The genetics of epigenetics

It is clear that epigenetic changes occur in cells as they age, and it appears plausible that such epigenetic changes contribute to the aging process. However, it is also interesting to notice that the rate of aging is quite distinct between genetically distinct individuals, or between different strains of mice (Yuan et al. 2009). Thus, a fascinating question in the field is whether it is possible that some of the differences in epigenetic marks that arise during stem cell aging are in fact controlled by genetic polymorphisms. Such polymorphisms could, for example, reside in loci encoding for the many epigenetic writers and erasers (thus affecting enzymatic activity) or in non-coding regulatory loci. Studies in humans have contributed to the identification of quantitative trait loci (QTLs) or polymorphisms that affect histone modifications or RNA polymerase II occupancy (McVicker et al. 2013; Grubert et al. 2015). As part of the Roadmap Epigenomics Consortium, the integrative analysis of 111 reference human epigenomes and the impact of DNA sequence and genetic variation on epigenomic state was investigated. From this analysis, it appeared that indeed histone modifications and DNA methylation can be predicted by the underlying DNA sequence using DNA motifs analysis in ES cells. Moreover, allelic bias in both transcript levels and epigenomic marks was found for each epigenome analyzed (Roadmap Epigenomics et al. 2015).

While studies from the ENCODE consortium were carried out in human cells, other mammalian models can also be used. For example, the use of genetically diverse inbred mouse strains with different lifespans allows quantitative trait locus analyses to determine the genomic locations of loci that are associated with the aging process. Early studies tested a subset of the BXD (C57BL/6J × DBA/2J) recombinant inbred panel of mice for lifespan (de Haan et al. 1998), and demonstrated that intra-strain variability of lifespan appeared to be genetically controlled. This suggested the presence of loci that induce individual variability among members of an inbred strain of mouse. Such loci would be predicted to encode for epigenetic modifiers, where polymorphisms would result in robust or disorganized epigenetic memory. More recently, other QTLs associated with aging in mice have been mapped (Klebanov et al. 2001; Miller et al. 2002). Therefore, it would be highly interesting to search for and identify the molecular nature of genetic loci that control the extent of epigenetic regulation in stem cells as they age.

## Perspectives

Aging is a complex process from which no living organism escapes. Identifying the molecular mechanisms that contribute to, if not cause, aging is a major challenge in aging research. The possibility that epigenetic alterations in aged stem cells contribute to aging of specific tissues and thus to organismal aging has received an ever-increasing attention, partly due to the appealing potential reversibility of such epigenetic modifications. A growing body of evidence, discussed in this review, has shown that epigenetic alterations accumulate as stem cells age, although most of these observations are often species or tissue specific. Substantial effort is required in order to identify whether a common epigenetic signature in aged stem cells exists. This is gradually becoming feasible due to technological advancements to screen multiple histone marks genome-wide. Critical in such analysis will be the use of highly purified and homogeneous cell populations, to rule out bias originating from cellular heterogeneity. In most tissues, purified stem cell populations are only available in limited quantities and therefore they have been so far excluded from genome-wide analyses that require high-input protocols. However, this will soon change as technological advancements will allow highly sensitive analyses using a low number of (stem) cells. It will be very interesting to address how the epigenome of a stem cell changed over its lifespan that is during aging. It will also be very important to standardize pipelines for the data analysis of genome-wide epigenetic data. Multiple complementary efforts have been undertaken by individual laboratories in understanding how the organization of the epigenome varies across rare cell types and different states, included aging. However, due to the early stage of low-input ChIP-seq assays and to the fact that often custom-made pipelines were used for the analysis of these datasets, the results are still in their infancy and are very difficult to be cross-compared between different laboratories. At the moment, it is not obvious that common epigenetic stem cell aging patterns can indeed be identified or rather whether aging at the epigenetic level is a completely random and unpredictable process. Either way, if epigenetic alterations can be causally linked to stem cell aging, targeting of such alterations in aged stem cells might be feasible in the near future. This would open the possibility to reverse, at least some, aging-associated deleterious epigenetic modifications.
